# The Correlation Between Vitamin D Levels and the Glycemic Marker HbA1c and Lipid Profile in Patients With Type 2 Diabetes Mellitus: A Study at the King Saud Medical City, Riyadh

**DOI:** 10.7759/cureus.57927

**Published:** 2024-04-09

**Authors:** Abeer B Alotaibi, Abdulrahman M.ELnasieh, Khalil Alduraibi

**Affiliations:** 1 Family Medicine Department, King Saud Medical City, Riyadh, SAU

**Keywords:** diabetes mellitus, glucose metabolism, lipid, sunlight, vitamin d

## Abstract

Background and objective

Vitamin D, a fat-soluble vitamin also called the sunshine vitamin, is produced in plants, and animals when exposed to sunlight. It plays a crucial role in musculoskeletal development, immune system regulation, and glucose metabolism, thereby reducing the risk of diabetes. This study aimed to investigate the association of vitamin D levels with glycemic control markers [glycated hemoglobin (HbA1c)] and lipid profile, as well as sociodemographic factors and comorbidities.

Methodology

A cross-sectional study was conducted at the King Saud Medical City in Riyadh, Saudi Arabia, among adult diabetic patients aged 20 years and above. The sociodemographic characteristics, vitamin D levels, HbA1c, and lipid profiles of 472 participants were studied. Data were analyzed using SPSS Statistics version 27 (IBM Corp., Armonk, NY).

Results

The majority of the participants were women (n=296, 62.7%); the mean age of the cohort was 56.5 ±13.13 years. Most participants were Saudi nationals (n=361, 76.5%). Lab tests revealed vitamin D deficiency (71.41 ±36.88 nmol/l) and elevated HbA1c (9.49 ±9.85%) in the cohort. Low-density lipoprotein (LDL) cholesterol levels were higher than normal (2.71 ±4.26 mmol/l), while high-density lipoprotein (HDL) was slightly lower (1.23 ±0.39 mmol/l). Bivariate correlations showed weak negative and positive associations between vitamin D and HbA1c (r=-0.093, p<0.05) and HDL (r=0.114, p<0.05), respectively. HbA1c correlated positively with triglycerides (r=0.168, p<0.01).

Conclusions

We found an association between deficiency of vitamin D and levels of HbA1c and lipid profile in type 2 diabetes patients. The association was marked by low vitamin D levels and characterized by high HbA1c, LDL cholesterol, and lipid profile. Elevated HbA1c, LDL cholesterol, and triglyceride levels suggested vitamin D's role in lipid homeostasis. Variations in biomarker levels across sociodemographic factors highlight the need for personalized interventions for diabetes prevention and management.

## Introduction

Vitamin D, a fat-soluble vitamin, is essential for various bodily functions in human beings. It can be produced in animals, fungi, and plant tissues when they are exposed to sunlight [1], and hence it is also known as the sunshine vitamin [2]. Due to the chemical nature of this compound, it is referred to as a steroid and a precursor molecule that can stimulate the production of human steroid hormones. The structure of one molecule of vitamin D consists of one side chain and four ring structures [3].

The major sources of vitamin D in humans are of two types: one which can be obtained from food and the other which is synthesized in the skin after exposure to sunlight. The sources of food rich in vitamin D, such as fish liver oil, contain ergocalciferol (D2), while dermal synthesis mechanisms provide cholecalciferol (D3) compounds. Both of these precursor compounds are processed in the human body by the liver enzyme 25-hydroxylase, which converts D2 into 25-hydroxyvitamin D2 (25-OH-D2) and D3 into 25-hydroxyvitamin D3 (25-OH-D3). Further catalysis is performed by kidney enzyme 25-hydroxyvitamin D-1 alpha-hydroxylase (CYP27B1), which turns both of these intermediate compounds into 1,25 dihydroxyvitamin D, which is the active form of vitamin D in the body [2,4].

The most recognized vitamin D functions in the human body pertain to its role in musculoskeletal development and functionality [2,5]. Vitamin D is directly involved in the absorption of phosphorus and calcium. The receptors for vitamin D are present on the nuclear surfaces inside the cells of tissues in organs of different systems including the digestive system (colon and small intestine), immune system (mononuclear cells, T lymphocytes, B lymphocytes), nervous system (brain) and musculoskeletal system (skin) [2,6]. These receptors bind to vitamin D molecules and activate or deactivate the transcription of a specific set of genes. The products of these genes stimulate phosphorus and calcium absorption in the digestive tract. The presence of sufficient amounts of vitamin D in the human body might increase the intestinal absorption of calcium from 10-15% to 30-40% and improve the intestinal absorption of phosphorus up to 80% [2,4].

Moreover, vitamin D also plays a significant role in modulating the mechanisms involved in various immune system-related abnormalities such as aggravated inflammatory responses and autoimmune disorders. It can also play a role in the downregulation of the processes involved in the adaptive immune system by producing an anti-inflammatory effect. Furthermore, vitamin D might also induce immunological tolerance in the human body [3]. Another significant role played by vitamin D is associated with the regulation of glucose metabolism and homeostasis. The secretion of insulin is regulated by vitamin D and can also interfere with the insulin sensitivity and maintenance of blood glucose levels [7]. Several studies have indicated the important link between vitamin D levels and its impact on reducing diabetes mellitus risk. It is speculated that there might be a close association between elevated vitamin D levels and improved regulation of glucose homeostasis in the human body. Hence, vitamin D can be considered as a protective compound to control autoimmune diabetes and regulate blood glucose levels in pre-diabetic patients as well as patients affected by type 1 and 2 diabetes mellitus [7,8].

The full-spectrum mechanistic details of vitamin D-associated regulation of glucose homeostasis are not fully understood yet. However, the role of vitamin D in genomic as well as non-genomic interactions with key regulators has been reported. Signaling molecules such as phosphatidylinositol-3 kinase, protein kinases A and C, phospholipase C, and mitogen-activated protein kinases (MAPK) are activated with the interactions of vitamin D. Also, several messenger molecules such as fatty acids and cyclic adenosine monophosphates (cAMP) have also been observed to be activated by the actions of vitamin D. Complex cascades of interactions mediated through receptors of vitamin D present in adipocytes and β-cells of pancreas have been studied to explore the relationship of vitamin D levels with glycemic control, insulin resistance, inflammation, and diabetes mellitus [9,10]. In light of this, this study aimed to investigate the relationship between vitamin D levels and the glycemic control marker [glycated hemoglobin (HbA1c)] and lipid profile, as well as sociodemographic factors and comorbidities among diabetic patients.

## Materials and methods

Study design and setting

This was a cross-sectional study conducted at the King Saud Medical City in Riyadh, Saudi Arabia from December 2022 to January 2023.

Eligibility criteria

The target study population was adult diabetic patients. The inclusion criteria were as follows: any adult diabetic patient aged >20 years presenting at the King Saud Medical City. However, any adult patient with gestational diabetes and children and adolescents aged less than 20 years were excluded from the study.

Sample size calculation

The sample size was calculated using the EPI info program. Based on a 95% confidence interval, 5% margin of error, and total population of adult diabetic patients aged >20 years presenting at the King Saud Medical City in Riyadh, Saudi Arabia. The estimated sample size was calculated to be 384 and was adjusted to 472 to compensate for an anticipated 10% non-response rate.

Data collection

The study involved collecting data in a data sheet from the Hospital files database, which contains information regarding the sociodemographic characteristics of the participants, such as age group, and gender. Furthermore, values regarding HbA1c level, lipid profile, and vitamin D levels of the patients were also obtained.

Sampling technique

A convenient non-probability sampling technique was employed to collect the data from the participants.

Statistical analysis

The statistical analysis involved both descriptive and inferential methods. Kolmogorov-Smirnov tests were used to assess normal distribution; all variables showed p-values <0.05, indicating non-parametric measures. Continuous variables were described using means with standard deviations (SD), while categorical variables were presented using frequencies and percentages. Spearman's rank correlation coefficient explored correlations among vitamin D, HbA1c, and lipid profile. The Mann-Whitney U test was employed to identify significant associations between lab markers and sociodemographic variables. Significance was determined at a p-value of 0.05 or lower, maintaining a 95% confidence interval. SPSS Statistics version 27 was used for all statistical computations (IBM Corp., Armonk, NY).

Ethical considerations

Approval for this study was granted by the Institutional Review Board (IRB) at the King Saud Medical City (Reference No: H1RI-24-Mar23-01, dated March 27, 2023). All data were kept confidential and used only for research purposes.

## Results

The study involved 472 participants, predominantly women (n=296, 62.7%) with a mean age of 56.5 ±13.13 years. More than three-quarters of the participants were Saudi nationals (n=361, 76.5%), with the remaining quarter representing various other nationalities, including India (n=19, 4%), Sudan (n=18, 3.8%), Yemen (n=14, 2.9%), the Philippines (n=12, 2.5%), and other countries (Table 1).

**Table 1:** The sociodemographic characteristics of the participants (n=472) SD: standard deviation

Variable	Values	
Age in years, mean (SD)	56.50 (13.13)	
Gender, n (%)	Female	296 (62.70)
	Male	176 (37.30)
Nationality, n (%)	Saudi Arabia	361 (76.5)
	Afghanistan	1 (0.2)
	Bangladesh	4 (0.8)
	Egypt	6 (1.3)
	Eritrea	4 (0.8)
	India	19 (4.0)
	Indonesia	2 (0.4)
	Jordan	1 (0.2)
	Niger	3 (0.6)
	Pakistan	8 (1.7)
	Palestine	2 (0.4)
	Philippines	12 (2.5)
	Senegal	1 (0.2)
	Sri Lanka	2 (0.4)
	Sudan	18 (3.8)
	Syria	6 (1.3)
	Tunisia	1 (0.2)
	Uganda	1 (0.2)
	Yemen	14 (2.9)
	Unknown	3 (0.6)

The collective lab tests for vitamin D, HbA1c, and lipid profile, encompassing low-density lipoprotein (LDL) cholesterol, high-density lipoprotein (HDL), triglycerides, and total cholesterol, were performed in all participants. The mean estimated vitamin D level was 71.41 ±36.88 nmol/l, indicating a relative deficiency given the normal threshold of 75 nmol/l or higher. HbA1c (normal range: 4.8-5.9%) averaged at 9.49 ±9.85%, exceeding the upper limit due to the participants' diabetic status. LDL cholesterol levels (normal: 2.59 mmol/l) showed a higher mean at 2.71 ±4.26 mmol/l. Similarly, triglycerides (normal: 1.70 mmol/l) displayed a slightly elevated mean among participants (1.79 ±1.21 mmol/l). HDL (normal: 1.68 mmol/l) showed a slightly lower mean among participants (1.23 ±0.39 mmol/l). Additionally, the estimated mean total cholesterol level fell within the normal range at 4.50 ±1.31 mmol/l (normal level: 5.2 mmol/l) (Table 2).

**Table 2:** Descriptive statistics of lab biomarkers HDL: high-density lipoprotein; LDL: low-density lipoprotein; SD: standard deviation

Variables	Normal level	Cohort value, mean (SD)
Vitamin D (nmol/l)	≥75	71.41 (36.88)
HbA1c (%)	4.8-5.9	9.49 (9.85)
LDL cholesterol (mmol/l)	<2.59	2.71 (4.26)
HDL (mmol/l)	>1.68	1.23 (0.39)
Triglycerides (mmol/l)	<1.70	1.79 (1.21)
Total cholesterol (mmol/l)	<5.20	4.50 (1.31)

Spearman's rank correlation coefficient was used for understanding correlations between biomarkers, revealing insights into potential health implications. We found a negative correlation between vitamin D and HbA1c (r=-0.093, p<0.05), indicating that higher Vitamin D levels tend to be associated with lower blood sugar levels. Additionally, vitamin D had a positive correlation with HDL (r=0.114, p<0.05), suggesting that higher vitamin D levels are linked with elevated levels of good cholesterol. However, significant correlations were not found between vitamin D and other biomarkers. Furthermore, a positive correlation was observed between HbA1c and triglycerides (r=0.168, p<0.01), indicating a potential relationship between higher blood sugar levels and increased triglyceride concentrations in blood. Moreover, LDL cholesterol showed positive correlations with several markers including HDL (r=0.132, p<0.01), triglycerides (r=0.173, p<0.01), and total cholesterol (r=0.895, p<0.01). These results suggested that increased levels of LDL cholesterol were associated with higher levels of HDL, total cholesterol, and triglycerides in the blood. Additionally, HDL exhibited a negative correlation with triglycerides (r=-0.387, p<0.01) and a positive correlation with total cholesterol (r=0.209, p<0.01), highlighting its role in maintaining a favorable lipid profile (Table 3).

**Table 3:** Bivariate correlations between all lab biomarkers **p<0.01; *p<0.05 (2-tailed) HDL: high-density lipoprotein; LDL: low-density lipoprotein

		1	2	3	4	5	6	7
1	Age	1						
2	Vitamin D (nmol/l)	0.152**	1					
3	HbA1c (%)	0.092*	-0.093*	1				
4	LDL cholesterol (mmol/l)	-0.256**	-0.065	-0.049	1			
5	HDL (mmol/l)	-0.110*	0.114*	-0.072	0.132**	1		
6	Triglycerides (mmol/l)	-0.039	-0.066	0.168**	0.173**	-0.387**	1	
7	Total cholesterol (mmol/l)	-0.282**	-0.046	-0.014	0.895**	0.209**	0.297**	1

Age showed significant correlations with all biomarkers except triglycerides. Positive correlations existed between age and vitamin D (r=0.152, p<0.01) and HbA1c (r=0.092, p<0.05) levels. Conversely, negative correlations were observed between age and LDL (r=-0.256, p<0.01), HDL (r=-0.110, p<0.05), and cholesterol levels (r=-0.282, p<0.01). As for gender, no significant differences were found regarding vitamin D and HbA1c levels. However, men exhibited higher LDL (2.89 ±6.84 mmol/l, p=0.045) and triglyceride (1.94 ±1.46 mmol/l, p=0.032) levels, while women had higher HDL (1.32 ±0.42 mmol/l, p<0.001) and cholesterol (4.64 ±1.31 mmol/l, p=0.002) levels. In terms of nationality, there were no significant differences in biomarker levels between Saudi and non-Saudi participants, except for HDL and triglycerides. Saudi participants showed higher HDL levels (1.26 ±0.41 mmol/l, p=0.011), while non-Saudi individuals had higher triglyceride levels (2.01 ±1.71 mmol/l, p=0.038) (Table 4).

**Table 4:** Variations in biomarkers values based on sociodemographic data HDL: high-density lipoprotein; LDL: low-density lipoprotein; SD: standard deviation

Variables	Gender		U-value	P-value	Nationality		U-value	P-value
	Male	Female			Saudi	Non-Saudi		
	Mean (SD)	Mean (SD)			Mean (SD)	Mean (SD)		
Vitamin D	69.03 (37.68)	72.84 (36.39)	23606.0	0.112	72.55 (37.43)	67.69 (34.94)	21743.0	0.119
HbA1C	10.34 (13.38)	8.98 (6.93)	25219.0	0.563	9.91 (11.19)	8.12 (1.82)	20904.5	0.489
LDL	2.89 (6.84)	2.59 (1.11)	23177.0	0.045	2.74 (4.83)	2.61 (1.10)	18676.0	0.279
HDL	1.09 (0.29)	1.32 (0.42)	15926.0	<0.001	1.26 (0.41)	1.14 (0.30)	23212.0	0.011
Triglycerides	1.94 (1.46)	1.70 (1.02)	29125.5	0.032	1.72 (0.99)	2.01 (1.71)	17422.5	0.038
Cholesterol	4.27 (1.28)	4.64 (1.31)	21585.5	0.002	4.48 (1.33)	4.57 (1.27)	19233.0	0.523

## Discussion

Our cohort's observed mean vitamin D level of 71.41 nmol/l indicated a relative deficiency, as it fell below the normal threshold of 75 nmol/l. This deficiency might contribute to challenges in glycemic control, evident in the elevated HbA1c levels averaging 9.49%. This aligns with a study conducted by Ozdin et al. (2023) [11], where diabetic patients exhibited significantly higher HbA1c levels (9.023 ±1.72%). This suggests a consistent association of diminished vitamin D levels with compromised glycemic regulation, with a notable 51% negative correlation [11].

Exploring lipid profiles, the current study identified elevated LDL cholesterol levels (2.71 mmol/l) and triglycerides (1.79 mmol/l), indicative of disturbances in lipid metabolism. The strong positive correlation between LDL cholesterol and total cholesterol levels underscores the interconnectedness of these lipid markers. A previous study by Dibaba (2019) [12] supports the current study findings, emphasizing the beneficial effects of vitamin D supplementation in reducing serum LDL cholesterol, total cholesterol, and triglyceride levels [12]. Specifically, a 69% decrease in triglyceride levels was observed post-supplementation.

Ghavam et al. (2018) [13] have provided insights into an inverse linear relationship between vitamin D levels and HbA1c, along with fasting blood sugar, further enriching our understanding [13]. The current study reflects this through a negative correlation of vitamin D with HbA1c (r=-0.093) and a positive correlation with HDL (r=0.114). These correlations are consistent with the findings of Buhary et al. (2017) [14], who reported a significant inverse relationship of HbA1c with vitamin D levels. They found that 73.1% of patients had 25(OH)D levels <50 nmol/L and observed a reduction in HbA1c from a mean of 10.55 to 7.70 following vitamin D supplementation. The observed inverse correlation of HbA1c with serum vitamin D levels in their study was robust, with a correlation coefficient of -0.14 (p<0.000) and -0.16 (p<0.000) pre- and post-vitamin D supplementation, respectively [14].

Sociodemographic factors were found to impact biomarker levels. Age emerged as a significant factor, demonstrating correlations with various biomarkers. Positive correlations of age with vitamin D (r=0.152) and HbA1c (r=0.092) levels indicated age-related variations in these parameters. Conversely, negative correlations of age with LDL (r=-0.256), HDL (r=-0.110), and cholesterol (r=-0.282) levels suggested age-related dynamics in lipid metabolism. These findings contrast with the findings of Elshebiny et al. (2021) [15], where cholesterol levels were observed to be higher among older patients [15], with a 32.3% prevalence of high cholesterol levels in the studied diabetic population.

Regarding gender, the current study found that men had increased bad cholesterol (LDL cholesterol) and triglyceride levels, while women had increased good cholesterol (HDL) and total cholesterol levels. However, there were no significant differences in vitamin D levels between both genders. Zhao et al. (2020) [16] have demonstrated that vitamin D deficiency is linked to higher HbA1c levels, especially in women [16]. The noteworthy findings, indicating a significant difference in HbA1c levels between vitamin D-deficient and non-deficient groups, revealed the relevance of gender-specific considerations [16]. Furthermore, to provide a perspective on gender-specific metabolic dynamics, future studies could explore the association between lipid profiles, vitamin D levels, and glycemic control across diverse populations.

The diversity of nationalities among the patients further contributed to the complexity of the current study. While no significant disparities were identified in biomarker levels between Saudi and non-Saudi participants, intriguing variations surfaced in HDL and triglycerides. Saudi participants exhibited higher HDL levels (mean: 1.26 mmol/l), while non-Saudi individuals had higher triglyceride levels (mean: 2.01 mmol/l). This coincides with the findings of Elshebiny et al. (2021) [15], where vitamin D levels varied between patients from Saudi Arabia and those from other countries [15], and revealed a notable 11.8% difference in average vitamin D levels between male patients from Saudi Arabia and those from other countries.

The current study findings underscore the significance of vitamin D in diabetes management and, potentially, cardiovascular health. The observed associations between decreased vitamin D levels and compromised glycemic control suggested that optimizing vitamin D might improve outcomes in diabetic individuals [17,18]. To complement the current study findings, relevant insights can be drawn from a study examining distinct vitamin D3 supplementation strategies in individuals with diabetic nephropathy [17]. The study shed light on the complex interplay between the status of vitamin D and bone health in individuals with diabetes [17]. Moreover, another study has emphasized the potential of optimizing the status of vitamin D in reducing type 2 diabetes risk [18].

While acknowledging the challenges in establishing causation due to the observational nature of cohort studies, ongoing randomized trials in well-defined populations, such as those with pre-diabetes, are expected to provide more conclusive evidence about the role of vitamin D supplementation in diabetes prevention [18]. These additional perspectives contribute to a more comprehensive understanding of the implications of vitamin D in diabetes management. Additionally, the positive link between vitamin D and HDL implies a potential preventive role in reducing adverse lipid profiles and reducing cardiovascular risk factors [19]. These implications highlighted the importance of considering vitamin D status in healthcare strategies.

Future research should explore the underlying mechanisms of the observed correlations, exploring molecular pathways to enhance the understanding of how vitamin D influences glycemic control and lipid metabolism [7,20]. Longitudinal studies need to be conducted to establish causality and investigate the sustained effects of maintaining optimal vitamin D levels on metabolic health [21]. Incorporating genetic factors into the analysis can provide insights into individual responses to vitamin D, paving the way for precision interventions. Additionally, exploring synergies between vitamin D and existing therapeutic approaches in diabetes management and cardiovascular health can guide holistic interventions for improved health outcomes [22].

This study has a few limitations. The employment of a cross-sectional study design hinders the establishment of causal relationships. Hence, further longitudinal studies are warranted to better understand the temporal dynamics of the observed correlations between the levels of vitamin D, glycemic control, lipid profiles, and sociodemographic factors. Additionally, the current study relied on self-reported sociodemographic information, introducing the potential for recall bias. Moreover, the study sample was drawn from a certain category of population with specific sociodemographic characteristics, which might limit the generalizability of findings to broader populations.

## Conclusions

We found a significant association between vitamin D deficiency and markers of glycemic control and lipid profile in type 2 diabetes mellitus patients. The observed vitamin D deficiency, coupled with elevated HbA1c, LDL, and triglyceride levels, suggests a potential role of vitamin D in regulating glucose metabolism and lipid homeostasis. The bivariate correlations further illuminate the intricate relationship of vitamin D with the key metabolic indicators. Moreover, this study unveiled the variations in biomarker levels across sociodemographic factors, with age, gender, and nationality exhibiting distinct correlations. The differential impact of vitamin D on these factors emphasizes the need for personalized interventions in managing diabetes-related complications. The current findings add to the growing body of knowledge on the multifaceted role of vitamin D in human health, particularly in the context of diabetes mellitus. Understanding these relationships can inform targeted strategies for both prevention and management, potentially reducing the burden of diabetes and its associated complications. Further research is warranted to delve deeper into the mechanistic aspects of these associations and explore potential avenues for therapeutic interventions aimed at optimizing vitamin D levels for improved metabolic health.
